# Single-cell mapping of focused ultrasound-transfected brain

**DOI:** 10.1038/s41434-021-00226-0

**Published:** 2021-02-01

**Authors:** A. S. Mathew, C. M. Gorick, R. J. Price

**Affiliations:** 1 Department of Biomedical Engineering, University of Virginia, Charlottesville, VA USA; 2 Department of Radiology & Medical Imaging, University of Virginia, Charlottesville, VA USA

**Keywords:** Neuroscience, Neurological disorders

## Abstract

Gene delivery via focused ultrasound (FUS) mediated blood-brain barrier (BBB) opening is a disruptive therapeutic modality. Unlocking its full potential will require an understanding of how FUS parameters (e.g., peak-negative pressure (PNP)) affect transfected cell populations. Following plasmid (mRuby) delivery across the BBB with 1 MHz FUS, we used single-cell RNA-sequencing to ascertain that distributions of transfected cell types were highly dependent on PNP. Cells of the BBB (i.e., endothelial cells, pericytes, and astrocytes) were enriched at 0.2 MPa PNP, while transfection of cells distal to the BBB (i.e., neurons, oligodendrocytes, and microglia) was augmented at 0.4 MPa PNP. PNP-dependent differential gene expression was observed for multiple cell types. Cell stress genes were upregulated proportional to PNP, independent of cell type. Our results underscore how FUS may be tuned to bias transfection toward specific brain cell types in vivo and predict how those cells will respond to transfection.

## Introduction

Despite increasing knowledge of the underlying mechanisms of many neurological diseases, safe and effective treatments are often lacking. Anatomical, physiological, and cellular obstacles make a therapeutic intervention in the central nervous system (CNS) extremely challenging. High vascularity and limited regenerative capacity of the CNS, along with the thickness and nonuniformity of the skull, significantly enhance the risk profile of any surgical approach. The blood-brain barrier (BBB), an arrangement of endothelial cells, tight junctions, basement membrane, astrocytic endfeet, and transport proteins common to most CNS vasculature, limits the vast majority of systemically injected therapies from accessing the brain [1]. Furthermore, current therapies for major neurological pathologies such as Alzheimer’s disease, Parkinson’s disease, and multiple sclerosis, are transiently effective and/or the only palliative. Thus, there exists a pressing need for the development of non-invasive, spatially-targeted, and durable treatment approaches across the spectrum of neurological disorders.

Focused ultrasound (FUS) mediated BBB disruption (BBBD) holds significant promise toward overcoming the aforementioned obstacles [2–4]. In this modality, gas-filled microbubbles (MB) and therapeutic agents are injected intravenously. Under image guidance, an extracorporeal transducer then directs conforming acoustic waves toward a pathologic region of the brain. These waves pass harmlessly through the skull and converge on the targeted region, causing the circulating MB to oscillate. These oscillations impart mechanical forces on cerebrovascular endothelium, temporarily disrupting BBB integrity and allowing therapeutics into the brain parenchyma. FUS mediated BBBD is targeted, non-invasive, and repeatable and has facilitated successful delivery of chemotherapies [5–7], antibodies [8–10], and even neural stem cells [11, 12].

Importantly, FUS BBBD also enables the delivery of systemically circulating gene therapies to the CNS [13–18]. Indeed, non-invasive gene delivery to the brain by FUS under precise image-guidance offers the prospect of curative therapies. However, translational hurdles still remain. First, knowledge of which brain-resident cell populations are most likely to be transfected after FUS-mediated BBBD and how transfection specificity depends on FUS parameters (e.g., PNP) are still unknown. Second, because the biophysical mechanisms through which gene delivery to the brain is achieved with FUS are complex, it is difficult to predict how FUS parameters like PNP will affect which cells are transfected and to what extent. Indeed, different brain cell types may exhibit markedly discrepant responses to FUS application and subsequent transfection. Recently, we used immunofluorescence analyses and single-cell RNA sequencing (scRNA seq) to determine that the specificity of transfection of endothelial cells of the BBB is inversely proportional to peak-negative pressure (PNP), a phenomenon we term “sonoselective” transfection [19]. Herein, we extend these previous scRNAseq studies considerably to investigate how the distribution of transfected brain-resident cell populations and their transcriptomes are affected by FUS PNP.

## Results

### FUS BBBD and brain cell transfection

**Fig. 1:**
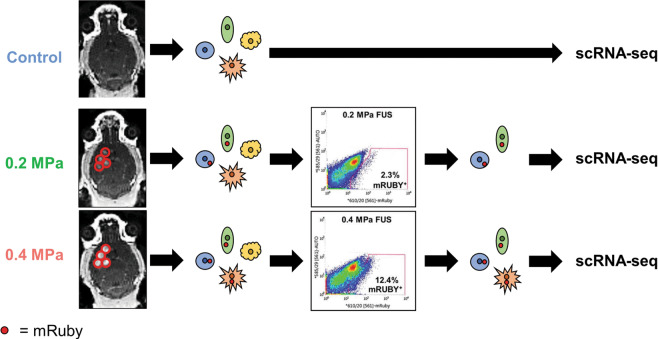
Overview of experimental methods. MR guided FUS was applied at either 0.2 MPa or 0.4 MPa to mouse striata following IV injection of mRuby plasmid conjugated to cationic MB. Brains were excised and dissociated, producing single-cell suspensions containing both untransfected and transfected cells. Using cells from the control condition to define the mRuby gating strategy, mRuby+ cells were sorted from FUS-treated brains by FACS. Single-cell RNA-sequencing was performed on untransfected, untreated cells from the control condition, mRuby+ cells from the 0.2 MPa condition, and mRuby+ cells from the 0.4 MPa condition.

Our experimental pipeline is shown in Fig. 1. Briefly, we intravenously injected cationic MBs and mRuby plasmid followed by MRI-guided FUS (1.1 MHz) targeted to the right striatum at either 0 MPa, 0.2 MPa, or 0.4 MPa PNP (estimated to be effectively 0 MPa, 0.164 MPa, and 0.328 MPa after skull attenuation). As expected, both MRI contrast enhancement in the targeted region and harmonic acoustic emissions were significantly greater at 0.4 MPa compared to 0.2 MPa (Fig. S1). After allowing 48 h for sufficient expression of mRuby by transfected cells, mouse brains were harvested and dissociated into single-cell suspensions. We then isolated live mRuby-expressing cells by fluorescence-activated cell sorting (FACS) and performed scRNA-seq. In total, 12.4% of dissociated cells treated at 0.4 MPa were mRuby^+^, compared to 2.3% treated at 0.2 MPa (Fig. 1). However, it is important to emphasize that we harvested the entire quadrant of the brain to ensure maximum cellular yield. Thus, these percentages are not representative of overall transfection efficiency. Given the weight of the harvested brains, the average density of the murine brain, and the volume of the −6 dB focal region for our transducer (i.e., 10.7 mm^3^), we estimate the true transfection efficiencies to be 28.5% and 5.4% at 0.4 MPa and 0.2 MPa, respectively. To establish the baseline proportions of brain-resident cell types and account for biases introduced in our dissociation protocol, cells from the 0 MPa treatment group were sequenced without mRuby FACS.

### FUS-transfected cell-type distributions depend on PNP

**Fig. 2:**
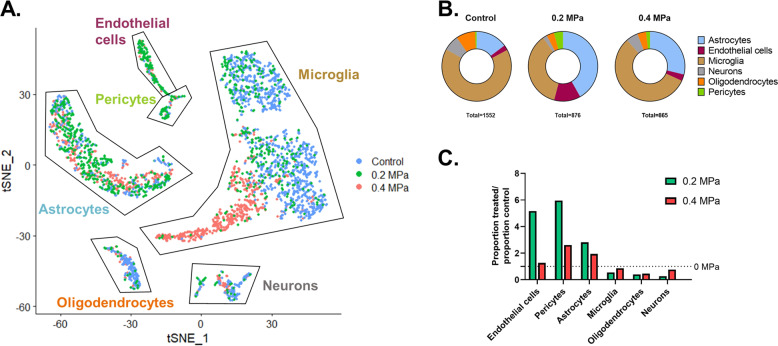
Identification of FUS-transfected cell types as a function of PNP. **A** t-SNE plot showing all sequenced cells, colored according to their treatment condition. Labels on the graph indicate cell populations identified by graph-based clustering followed by the analysis of globally distinguishing transcripts within each cluster. **B** Proportions of each of the six identified cell types for each condition. The total numbers of cells analyzed are shown below each chart. **C** Bar graph illustrating the influence of FUS PNP on the distribution of transfected cells (color figure online).

To assign cell identities to our dataset, we performed graph-based clustering followed by the comparison of globally distinguishing genes within each cluster against scRNA-seq databases. After filtering ambiguous clusters and pooling those of the same class, we identified six distinct cell types, namely astrocytes, endothelial cells, microglia, neurons, oligodendrocytes, and pericytes (Fig. 2A). The proportions of these mRuby+ cell types were dependent on PNP (Fig. 2B). Specifically, 0.2 MPa FUS transfection led to marked enrichment of cells comprising and in contact with the BBB (i.e., endothelial cells, pericytes, and astrocytes) compared to control, while 0.4 MPa FUS led to a transfection distribution in between that of 0.2 MPa transfection and 0 MPa controls (Fig. 2C). Thus, cells of the BBB (i.e., endothelial cells, pericytes, and astrocytes) are relatively enriched at lower FUS PNP while those farther from the BBB (neurons, oligodendrocytes, and microglia) are more efficiently transfected at higher FUS PNP.

### Transcriptional responses of individual FUS-transfected cells

**Fig. 3:**
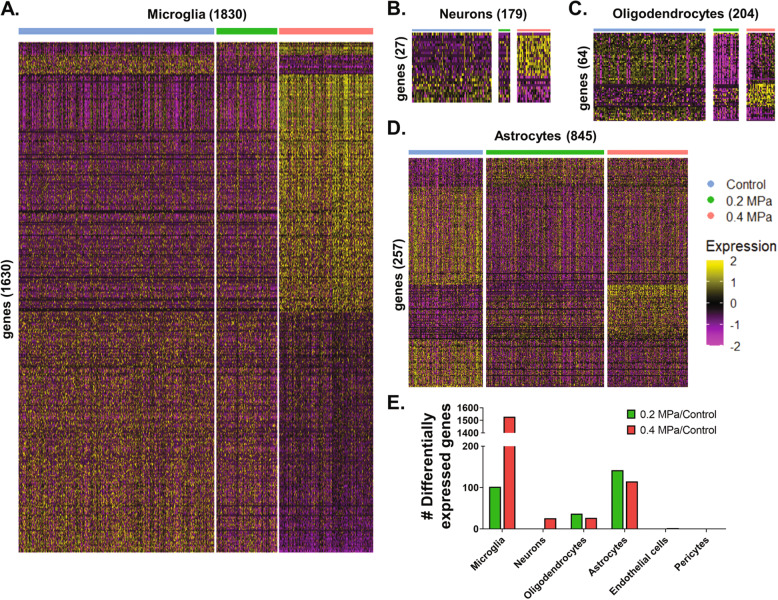
Transcriptional responses of individual-focused ultrasound-transfected cells. **A**–**D** Gene expression heatmaps for (**A**) microglia, (**B**) neurons, (**C**) oligodendrocytes, and (**D**) astrocytes. Each column represents a single-cell and each row represents a gene of interest. Selected genes for each cell type are significantly (*p*-adjusted < 0.05) upregulated or downregulated at 0.2 MPa or 0.4 MPa compared to control. Expression levels are presented as row-normalized *z*-scores according to the key. Numbers in parenthesis indicate the total number of cells (columns) or genes (rows) presented. **E** Magnitude of significant (*p* adjusted < 0.05) differential gene expression (upregulated + downregulated) for each cell type at each pressure vs control cells.

**Fig. 4:**
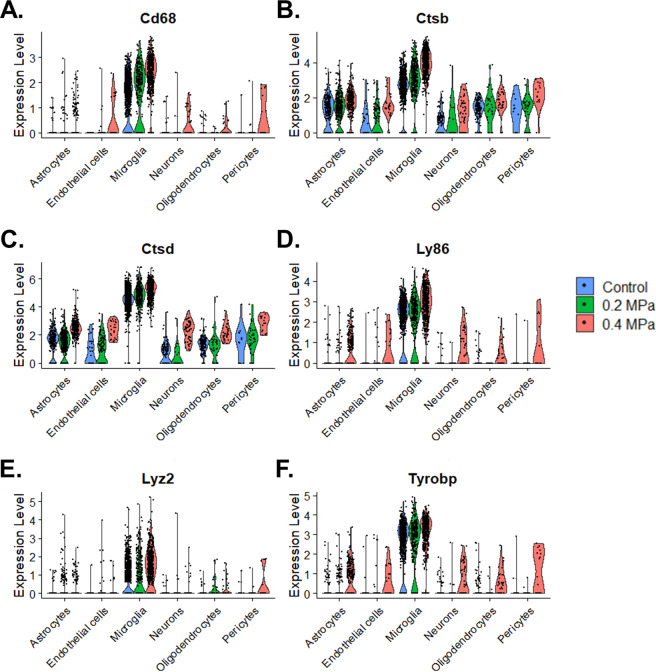
Genes associated with cell stress are upregulated across multiple cell types as a function of FUS PNP. **A**–**F** Violin plots of normalized expression levels for selected transcripts. Each dot represents a single-cell, grouped by cell type and treatment condition.

To assess cell-type-specific transcriptional responses to FUS-mediated BBBD and transfection, we performed differential expression testing, comparing 0.2 MPa and 0.4 MPa transfected cells to matching populations from the 0 MPa control group across multiple cell types (Fig. 3A–D). Transfected microglia exhibited massive differential gene expression (1630 significantly regulated transcripts) when compared to 0 MPa control cells, with 0.4 MPa PNP FUS exerting a much stronger effect than 0.2 MPa PNP FUS (Fig. 3A, E). While neurons exhibited the same PNP-dependent response, far fewer differentially regulated transcripts were identified overall (Fig. 3B, E). In contrast, neither oligodendrocytes (Fig. 3C, E) nor astrocytes (Fig. 3D, E) differentially expressed more transcripts at the higher PNP (i.e., 0.4 MPa). Overall, our results indicate that the absolute numbers and identities of significantly differentially expressed genes depend on cell type and FUS PNP (Fig. 3E and Supplemental Table 1). Finally, despite the robust cell type-specific responses shown in Fig. 3, we questioned whether there might exist sets of genes that are affected by FUS regardless of the cell type. Interestingly, careful curation of our dataset revealed that several genes associated with cellular stress and inflammation, including *CTSD, CTSB, LY86, CD68, LYZ2*, and *TYROBP*, are indeed significantly upregulated in multiple cell types as a function of increasing PNP (Fig. 4). A complementary analysis revealed *CKB, DNAJA1, HBB-BS, HSPA8, JUN, JUND, and RPS27* were downregulated across multiple cell types with increasing PNP (Fig. S2).

## Discussion

Conventional approaches for gene delivery to the CNS can be limited by their invasiveness, poor localization, systemic toxicity, or inefficient transit across the BBB. FUS activation of systemically administered MB surmounts all of these, as it is noninvasive, targeted, safe, and transiently disrupts the BBB [20]. While we and others have established the potential of this technology for gene therapy [13–18], considerable knowledge gaps still exist. Indeed, we reason that acquiring a more comprehensive understanding of (i) how FUS parameters affect which cell types are transfected and (ii) how these cells respond to transfection at the transcriptional level will permit fine-tuning of FUS-mediated transfection approaches for selected applications. Toward this end, we used scRNA-seq to quantify proportions of brain-resident cell types transfected by FUS, their transcriptional responses 48 h post-treatment, and the relationship of these metrics to PNP. Both 0.2 MPa and 0.4 MPa FUS application elicited successful transfection of endothelial cells, astrocytes, pericytes, neurons, oligodendrocytes, and microglia. While 0.2 MPa PNP preferentially transfected BBB-associated cells (i.e., endothelial cells, astrocytes, and pericytes), 0.4 MPa PNP shifted transfected cell-type distributions to include more microglia, neurons, and oligodendrocytes. These data, in conjunction with prior histological studies demonstrating that 0.1 MPa PNP is highly selective for endothelial cell transfection [19], are consistent with the hypothesis that the probability of a cell being transfected by FUS is directly proportional to PNP and inversely proportional to the distance from the microcirculation. Moreover, at least in the context of FUS transfection, our results suggest that any cell-type differences in transfection potential that may exist appear to be overridden by physical factors. While the extent and nature of significant differential gene expression were cells- and PNP-dependent, we identified several cellular stress-associated genes that were consistently upregulated independent of cell type and proportional to PNP. Together, these results provide high-resolution insight into the cellular implications of FUS mediated transfection that will ultimately refine the preclinical design and accelerate clinical translation.

Our experimental and computational pipeline enabled unbiased identification of six brain-resident cell types in the neurovascular unit (NVU). We noted a bias toward transfection of cells closer to the microcirculation, such as endothelial cells, astrocytes, and pericytes, especially at lower FUS PNP. Neurons, oligodendrocytes, and microglia were enriched with higher PNP, presumably because of enhanced plasmid availability beyond the BBB. Microglial activation in the context of PNP-dependent sterile inflammation may also lead to chemotaxis to the BBB, thereby increasing microglial propensity for transfection. Overall, our results are in agreement with previous work from our group, wherein gene-bearing nanoparticles were delivered instead of plasmid [21]. In that study, we observed higher transfection of astrocytes compared to neurons by immunofluorescence. Our model is also consistent with work in which FUS mediated delivery of recombinant adeno-associated virus (rAAV) elicited transduction of significantly more astrocytes than neurons [22]. However, we note disagreement with another rAAV study, which transduced primarily neurons [14]. This discrepancy could be attributed to differences in cellular uptake, expression stability for FUS-enhanced delivery of bacterial vs viral vectors, or FUS experimental parameters. Other studies of FUS-mediated viral gene delivery that demonstrate highly selective neuronal transgene expression utilize neuron-specific promoters [16]. Indeed, the overall approach and results presented here may be especially useful for choosing FUS parameters that best synergize with gene therapy approaches that utilize cell-specific promoters by biasing plasmid delivery to the cell type(s) of interest. Furthermore, independent of the specific gene delivery vehicle that is chosen for FUS transfection, our study provides a framework for how scRNA seq can be used to inform and optimize the transfection of selected cell types in the brain.

Several genes associated with cellular stress and inflammation were upregulated across multiple cell types in proportion to PNP. While many studies have demonstrated that FUS-mediated BBBD results in minimal damage at the tissue level [3, 23, 24], impacts at the cellular and molecular levels are actively under investigation. Transcriptomic and proteomic profiling by multiple groups has found that, under certain FUS and MB conditions, FUS mediated BBBD may elicit a sterile inflammatory response in the brain parenchyma [19, 25–27]. The precise mechanistic relationship between FUS-mediated BBBD and sterile inflammation remains unclear. Possible causes include direct acoustic damage to BBB, NVU injury caused by cavitation-induced shockwaves, ischemia-reperfusion injury caused by transient vasospasm, and exposure of the brain parenchyma to blood products. Sonoporation, one of the mechanisms by which FUS is proposed to enhance gene delivery, has been shown to generate large irreversible pores, increase reactive oxygen species, reduce endoplasmic reticulum mass, increase apoptosis, and delay the cell cycle [28–30]. It is probable that multiple interactions contribute to sterile inflammatory response induced by FUS. Given that we harvested tissue 48 h post-FUS to allow time for sufficient transgene expression, the differential gene expression profile we report is consistent with a landscape of resolving inflammation. We noted pressure-dependent upregulation of *CTSD*, *CTSB, LY86*, *LYZ2, CD68*, and *TYROBP* across multiple cell types. Cathepsin D, the protein product of *CTSD*, is a protease expressed in the lysosome involved in antigen processing, apoptosis, and biomolecule degradation [31, 32]. Studies of its role in Alzheimer’s disease suggest it is upregulated during neuronal repair [33]. Cathepsin B, another lysosomal protease, is activated in response to diverse inflammatory stimuli in multiple brain cell types and contributes to programmed cell death [34, 35]. The function of LY86 is not well understood, though it is thought to play a role in regulating inflammation and toll-like receptor (TLR) signaling [36, 37]. CD68 is a lysosomal protein that is upregulated in actively phagocytosing microglia [38]. While its expression was clearly the highest in microglia, we observed PNP dependent upregulation in all cell types. Non-myeloid expression of CD68 has been reported before as evidence of increased lysosomal activity [39]. Further evidence of microglial activation is supported by the PNP-dependent upregulation of *LYZ2* (Lysozome 2), a powerful antimicrobial hydrolase. Increases in *LYZ2* across multiple cell types were also observed in a scRNA-study of Niemann-Pick disease, a neurodegenerative pathology characterized by inappropriate activation of innate immunity [40]. Similarly, TYRO protein tyrosine kinase binding protein (TYROBP, the protein product of *TYROBP*) is also primarily expressed in microglia. TYROBP has complex functions in microglia, having roles in increasing phagocytic activity and decreasing cytokine production [41]. Non-myeloid expression of TYROBP has also been linked to neuroinflammation [42]. Interestingly, many of the genes highlighted by our analysis exactly match those found in a gene cluster specific to the resolution of neuroinflammation [43]. Notably, we did not detect significant upregulation of classical markers of sterile neuroinflammation such *as AIF1* in microglia, *GFAP* in astrocytes, and *ICAM1* in endothelial cells. Thus, our differential expression analysis is consistent with a resolving PNP-dependent inflammatory response 48 h post-FUS.

There are some limitations of this investigation. The requirement for dissociation of treated tissue to viable single-cell suspensions and myelin removal prior to scRNA-seq likely limited the yield of large complex cells such as neurons or oligodendrocytes. We corrected for this methodological limitation by making comparisons to sequences from non-transfected cells that were subject to the same isolation methods. Nonetheless, while this approach does allow us to make relative comparisons, we are not able to accurately report the absolute extent of transfection on a per-cell-type basis without making significant assumptions. Further, the process of mechanical and enzymatic dissociation itself may have imparted transcriptional effects on the sequenced cells. Finally, due to the high processing complexity and cost of scRNA-seq, replicates were not sequenced separately. Instead, we pooled multiple biological replicates from each condition prior to FACS and scRNA-seq library preparation and subsequently ran all samples in the same sequencing run. This approach is common [44, 45] and has been shown to mitigate batch effects and improve statistical power [46, 47].

To summarize, we used single-cell RNA-sequencing to study the effects of 0.2 MPa and 0.4 MPa FUS-mediated transfection on the brain. At 48 h post-treatment, we observed lower overall transfection at 0.2 MPa compared to 0.4 MPa, but higher selectivity for cells comprising the BBB, namely endothelial cells, astrocytes, and pericytes. Differential gene expression analysis highlighted PNP dependent, cell-type independent upregulation of genes associated with cellular stress. This work has significant implications for the design of future investigations leveraging FUS-mediated transfection. For applications where higher cell-type specificity and/or lower cellular stress are required, lower PNPs should be used. Inversely, for applications where higher general transfection is desired, and when a sterile inflammatory response is tolerable (or even desirable), higher PNPs may be recommended. Other FUS experimental parameters (such as frequency, pulsing interval, duty cycle, burst length, and MB dose) are also likely to affect transfection selectivity and efficiency and could be tested in future investigations.

## Methods

The work presented herein is an extended analysis of a dataset generated in previous studies by our group [19]. For the reader’s edification, the experimental methods employed to generate the scRNA seq dataset are provided in the Supplemental Information.

### Single-cell RNA sequencing and analysis

After FACS, 0 MPa (unsorted), 0.2 MPa (mRuby^+^), and 0.4 MPa (mRuby^+^) single-cell libraries were generated using the chromium controller (10X Genomics, Pleasanton, CA) with the chromium single-cell 3′ GEM, Library & Gel Bead Kit v3 (10X Genomics) and chromium single-cell B chip kit (10X Genomics). An average of 1482 cells per condition was sequenced on a NextSeq 500 (Illumina) at an average depth of 92,409 reads per cell. The CellRanger v3.0.2 pipeline was implemented to first convert bcl2 reads to FASTQ files followed by alignment to the mm10 (Ensembl 84) mouse reference genome and filtering. All further single-cell analysis was performed in R using Seurat v3.1.5 [48] with default parameters unless otherwise specified. Cells with low read depth, low expression diversity, or high mitochondrial content were filtered out of the analysis. Cell clusters were computed by graph-based clustering and subsequently identified by comparing the top 20 globally distinguishing markers (i.e., those with *p* adjusted <1E−240, average natural log fold change above all other cell types >0.25, and expressed in at least 25% of that cell type) with those having high cell-type specificity scores in the PanglaoDB web server [49]. Clusters of the same cell type were merged. Cells of unclear significance in the context of FUS mediated transfection including, ependymal cells, choroid plexus cells, and peripheral leukocytes were removed from the analysis. Differential gene expression between endothelial subsets was performed using the MAST framework [50]. PNP-dependent, cell-type independent genes were defined as those differentially regulated in at least 5/6 cell types at 0.4 MPa vs control with a *p*-value < 0.15.

## Supplementary information

Supplemental Material

## Data Availability

scRNA-seq data have been deposited in the Gene Expression Omnibus database (https://www.ncbi.nlm.nih.gov/geo/query/acc.cgi?acc=GSE%20141922).
