# Supplementary material for: Single-cell mapping of focused ultrasound-transfected brain
Source: Gene Ther. 2021 Feb 1;30(3-4):255–63. doi: 10.1038/s41434-021-00226-0 (PMC8325700; doi:10.1038/s41434-021-00226-0)
Supplement: Supplementary file 1 — Supplemental Material [file 41434_2021_226_MOESM1_ESM.pdf]

Figure S1

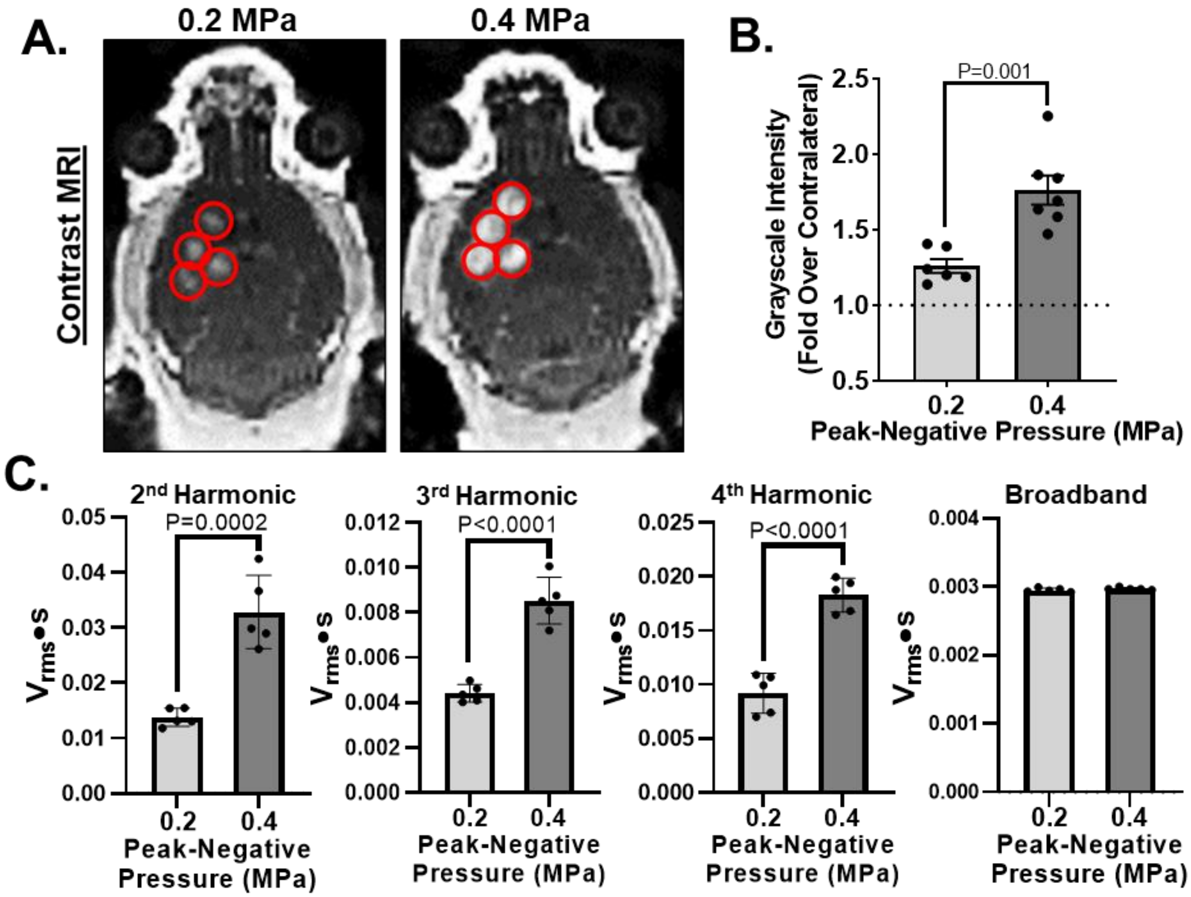

Figure S1. **Characterization of blood-brain barrier opening with focused ultrasound. A)** Contrast MR images of mouse brains after application of pulsed FUS in the presence of systemically administered MBs. FUS was applied at peak-negative pressures of 0.2 and 0.4 MPa, with a 4-spot sonication pattern. Sonication sights are denoted with red circles. **B)** Bar graph of contrast enhancement over contralateral FUS$^-$ control hemisphere as a function of pressure. **C**) Passive cavitation analyses for 2nd, 3rd, and 4th harmonics, as well as broadband emissions. All statistical comparisons by unpaired t-tests. Adapted with permission from Gorick et al. (*Proc Nat Acad Sci.* 117(11):5644-5654).

Figure S2

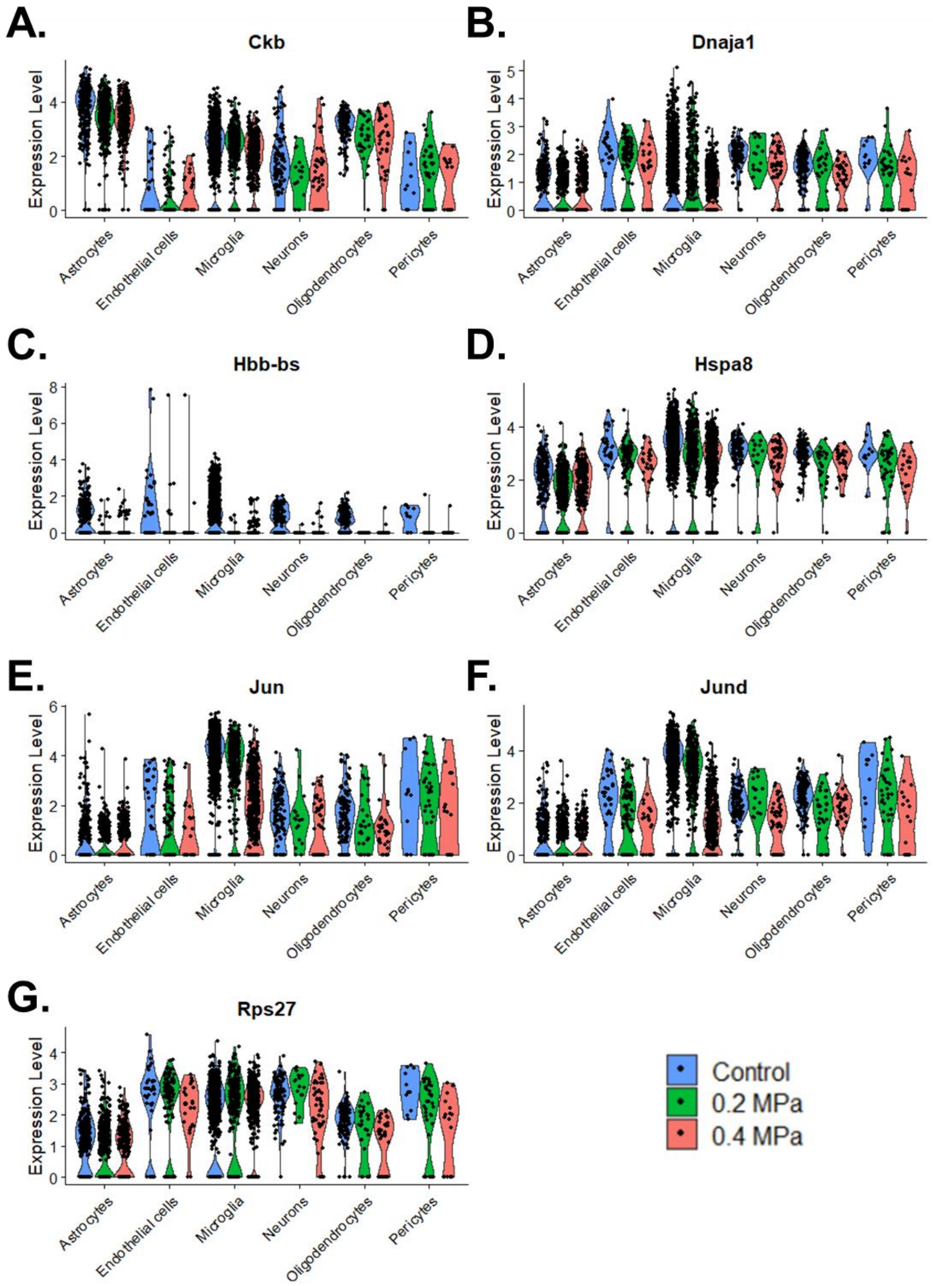

Figure S2. Genes downregulated across multiple cell types as a function of FUS PNP. A-G) Violin plots of normalized expression levels for selected transcripts. Each dot represents a single cell, grouped by cell type and treatment condition.

Figure S3

A.

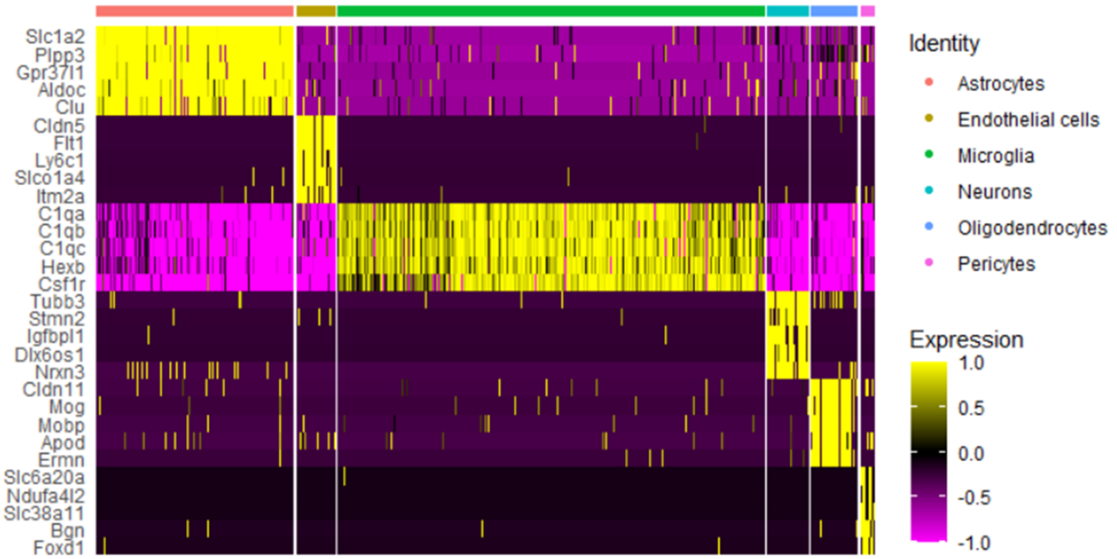

B.

Rank	Astrocytes	Endothelial cells	Microglia	Neurons	Oligodendrocytes	Pericytes
1	Slc1a2	Cldn5	C1qa	Tubb3	Cldn11	Slc6a20a
2	Plpp3	Flt1	C1qb	Stmn2	Mog	Ndufa4l2
3	Gpr37l1	Ly6c1	C1qc	Igfbpl1	Mobp	Slc38a11
4	Aldoc	Slco1a4	Hexb	Dlx6os1	Apod	Bgn
5	Clu	Itm2a	Csf1r	Nrxn3	Ermn	Foxd1
6	Atp1a2	Pltp	Ctss	Dcx	Enpp2	Mir143hg
7	Gja1	Ly6a	Ctsd	Dpysl3	Tspan2	Tbx18
8	Bcan	Spock2	Atf3	Stmn3	Stmn4	Ogn
9	Slc4a4	Cxcl12	Selplg	Dlx1	Opalin	Vtn
10	Mt3	Ptprb	Cx3cr1	Celf4	Ppp1r14a	Enpep
11	Htra1	Egfl7	Tmem119	Cd24a	Tubb4a	Pcolce
12	Cspg5	Abcb1a	Fcrls	Gad2	Ugt8a	Col3a1
13	Ttyh1	Igfbp7	Lgmn	6330403K07Rik	Pllp	Gpc3
14	Nrxn1	9430020K01Rik	Trem2	Bcl11a	Grb14	S1pr3
15	Sparcl1	Pglyrp1	P2ry12	Nsg1	Aspa	Ace2
16	Fjx1	Adgrf5	Tyrobp	Gad1	Sez6l2	Kcnj8
17	Ptprz1	Fn1	Junb	Sp9	Tmem151a	Higd1b
18	Slc6a11	Pcp4l1	Fcer1g	Bex2	Tmeff2	Twist1
19	Ndrg2	Adgrl4	Mafb	Dlx2	Edil3	Abcc9
20	Ntsr2	Sema3c	Laptm5	Nsg2	Efnb3	Aspn

Figure S3. Globally distinguishing transcripts used to assign cell-types to clusters. A) Gene expression heatmap showing the top 5 globally distinguishing genes from each cluster. Each row represents a single gene and each column represents a single cell, with each element of the heatmap representing a row-normalized expression value. Cluster membership is represented by the bars above the heatmap. **B)** The top 20 globally distinguishing transcripts from each cell cluster were compared against the PangloDB webserver[4] to assign cell type. Globally distinguishing transcripts were defined as very significantly (p adjusted < 1E-240), upregulated (average natural log fold change above all other cell types > 0.25), and expressed in at least 25% of that cell type.

Supplemental Table

Table S1: Top 25 most significantly differentially expressed genes at 0.2 MPa and 0.4 MPa compared to control for each cell type.

**Microglia**						**Astrocytes**					
**0.2 MPa**			**0.4 MPa**			**0.2 MPa**			**0.4 MPa**		
**symbol**	**avg_logFC**	**p_val_adj**	**symbol**	**avg_logFC**	**p_val_adj**	**symbol**	**avg_logFC**	**p_val_adj**	**symbol**	**avg_logFC**	**p_val_adj**
Hbb-bs	-1.7106704	1.10E-115	Jund	-2.12966211	3.26E-294	Hbb-bs	-1.29153602	1.15E-66	Hbb-bs	-1.16972319	9.85E-41
Hba-a1	-0.89951604	2.79E-36	Ctsb	1.27546198	1.80E-206	Hba-a2	-0.46252228	9.26E-23	Tmsb4x	0.76890122	1.05E-33
Cd81	-0.47836238	3.75E-33	Selplg	-1.1130985	1.27E-204	Hba-a1	-0.62888017	4.37E-22	Ctsd	0.85006323	1.70E-33
Hba-a2	-0.74332008	1.59E-27	Ier5	-1.49685688	1.07E-203	Hbb-bt	-0.52542572	1.14E-15	Ly86	0.76467565	1.68E-24
Hbb-bt	-0.5925249	2.30E-21	Jun	-1.56760745	7.30E-189	PISD	0.49188835	3.20E-13	Tyrobp	0.81617834	5.14E-23
Tmem176b	-0.6174352	2.40E-20	Junb	-1.58459307	3.85E-187	Dbi	-0.68587095	2.21E-12	C1qb	0.70061823	1.80E-22
Cd83	-0.66123733	1.44E-19	Ubc	-1.36825621	2.34E-180	H3f3b	-0.61372328	9.65E-12	Ank2	0.4715101	5.25E-13
Tmem119	-0.4634066	1.48E-18	Rpsa	1.11805242	1.39E-158	Ubb	-0.60309423	1.08E-11	Fcrls	0.5707902	1.59E-12
Ctsb	0.57018413	4.58E-18	Csf1r	-0.95354205	4.43E-157	Eif1	-0.54712225	1.09E-10	Gria2	0.48559285	1.67E-12
Atf3	-0.5666306	4.15E-17	Rps2	1.14592479	9.34E-153	Fabp7	-1.40576028	2.92E-10	Lgmn	0.58716787	1.75E-12
C1qa	-0.28208189	6.83E-17	Rhob	-1.11435431	3.50E-151	Nkain4	-0.62169416	3.63E-08	Hexb	0.52641333	2.04E-12
C1qc	-0.26199382	1.29E-14	Atf3	-1.55530981	4.11E-144	Grin2c	0.56944976	5.96E-08	Ubb	-0.6287844	1.83E-11
Selplg	-0.28300175	6.99E-13	Rps19	1.15345191	8.22E-143	Mpc1	-0.54014168	8.81E-08	Mt3	-0.69684725	2.00E-11
Ier5	-0.42126815	3.13E-12	Prdx1	1.25606692	1.23E-141	Hspa8	-0.46400036	9.88E-08	C1qa	0.54858542	2.31E-11
Ier2	-0.63172618	5.31E-12	Unc93b1	-0.94088107	4.16E-140	Cd9	-0.6097746	1.33E-07	Macf1	0.28370792	1.03E-09
Ubc	-0.38702291	5.32E-12	Btg2	-1.50503967	6.74E-139	Ppia	-0.48094134	2.13E-07	mt-Nd4l	0.4583164	1.24E-09
Rpl35	0.41977276	8.88E-12	Tmem119	-1.06037989	1.03E-138	Mertk	0.51127802	2.37E-07	Ctsb	0.45341917	1.47E-09
Tmem176a	-0.5731051	1.01E-11	Rpl32	1.17058763	4.38E-137	Vcl	0.463661	2.44E-07	Hba-a1	-0.55144011	1.55E-09
Cd68	0.35321071	4.34E-11	Rps20	1.10234054	2.25E-136	Prdx1	-0.52152968	5.63E-07	Grin2c	0.57839114	1.62E-09
Junb	-0.39761693	1.12E-10	Cd83	-1.65294619	5.76E-136	Prodh	0.46056433	5.69E-07	Mt2	-0.64971016	1.98E-09
C1qb	-0.26205448	1.14E-10	Mif	1.3893888	9.98E-135	Nrxn1	0.28030098	7.73E-07	C1qc	0.5328327	2.69E-09
Jund	-0.34436837	1.53E-10	H3f3b	-1.21914785	1.89E-134	Fez1	-0.49848005	9.95E-07	Hbb-bt	-0.49419105	1.11E-08
Rps28	0.37702417	2.47E-10	Hbb-bs	-1.60912711	2.07E-128	Acsl3	0.33944266	1.12E-06	Ldhb	-0.57952084	1.36E-08
Rps26	0.37806447	4.25E-10	Lgals1	1.65736327	9.25E-127	Rpl8	-0.47429529	1.16E-06	Nrxn1	0.34905109	2.05E-08
Actg1	-0.45536277	4.39E-10	Rpl14	1.00307058	1.68E-121	Chchd2	-0.45919547	1.16E-06	Nkain4	-0.69984864	6.45E-08
**Oligodendrocytes**						**Neurons**					
**0.2 MPa**			**0.4 MPa**			**0.2 MPa**			**0.4 MPa**		
**symbol**	**avg_logFC**	**p_val_adj**	**symbol**	**avg_logFC**	**p_val_adj**	**symbol**	**avg_logFC**	**p_val_adj**	**symbol**	**avg_logFC**	**p_val_adj**
Hbb-bs	-0.88213465	1.38E-13	Hbb-bs	-0.78490253	8.36E-11	Hbb-bs	-0.97678324	3.80E-03	Ctsd	1.49075697	3.44E-19
Trf	-0.91572771	7.59E-08	Ctsd	0.86076288	4.60E-09				Hbb-bs	-0.81298249	7.19E-08
PISD	1.89894975	5.33E-07	C4b	1.72161692	7.53E-09				Ly86	1.01968072	8.03E-08
Ubb	-0.54504629	2.68E-05	Serpina3n	1.39453378	8.68E-09				Cst3	0.66972402	3.81E-07
Fth1	-0.76783415	4.18E-05	Endod1	0.66454782	6.14E-08				Gm42418	0.89058893	2.18E-06
Slc48a1	-0.78395	5.90E-05	Plekha1	0.99470796	1.42E-06				C1qa	0.82210151	3.12E-05
Stmn4	-0.79281369	1.39E-04	Ubb	-0.41770552	1.81E-05				Actg1	-0.82547073	7.40E-05
Car2	-0.96981599	5.52E-04	Gstm5	-0.7261072	9.19E-05				C1qb	0.87732951	9.95E-05
Qdpr	-0.85502048	6.70E-04	Fabp5	1.21698285	3.40E-04				Lgmn	0.75816036	6.95E-04
Ptma	-0.60691154	9.50E-04	Mat2a	0.60974994	3.95E-04				Gnb1	-0.36605323	1.17E-03
Gatm	-0.72128819	9.75E-04	Ank3	0.77488641	4.36E-04				Tyrobp	0.82393792	2.51E-03
Sec11c	-0.73059016	1.31E-03	Stmn4	-0.59369992	4.53E-04				Ptms	-0.56245225	4.96E-03
Csrp1	-0.92714479	1.41E-03	Egr1	-1.06425312	2.16E-03				Ube3c	0.61336503	5.61E-03
Cryab	-0.80488817	2.47E-03	Csrp1	-0.60759123	3.53E-03				Fcrls	0.77190779	7.48E-03
Hsp90aa1	-0.54641709	3.64E-03	Dlk2	0.3323742	3.57E-03				Hbb-bt	-0.3427697	7.90E-03
Sez6l2	-0.66418779	4.20E-03	Tubb3	0.90953719	5.58E-03				mt-Co1	0.65613358	8.02E-03
Itm2b	-0.40977639	4.88E-03	Tulp4	0.58597021	6.78E-03				Jund	-0.66011931	9.10E-03
Cox4i1	-0.50577426	5.67E-03	Slc9a3r2	0.6952904	9.37E-03				Trpm7	0.97176787	1.01E-02
Igsf8	0.4524799	8.36E-03	Fez1	-0.50518529	1.27E-02				Ctsb	0.7690223	1.54E-02
Psmb7	-0.55280556	9.88E-03	Rps27	-0.53595975	1.55E-02				2610524H06F	-0.38794939	1.97E-02
Jund	-0.64035694	1.05E-02	Fcrls	0.58202078	2.30E-02				Dynll1	-0.54678969	2.53E-02
Efhd1	-0.57352124	1.18E-02	Cd63	0.97580669	2.30E-02				Cct8	-0.29753548	2.58E-02
Rabac1	-0.48352384	1.26E-02	Gadd45b	1.35715649	3.15E-02				6330403K07F	-0.46821657	3.01E-02
Eif1	-0.49575777	1.31E-02	Clock	0.53064675	3.30E-02				Gnai2	-0.70361002	3.04E-02
H3f3b	-0.50576516	1.66E-02	Actb	-0.42862232	3.98E-02				Cep350	0.47164902	3.44E-02
**Endothelial cells**						**Pericytes**					
**0.2 MPa**			**0.4 MPa**			**0.2 MPa**			**0.4 MPa**		
**symbol**	**avg_logFC**	**p_val_adj**	**symbol**	**avg_logFC**	**p_val_adj**	**symbol**	**avg_logFC**	**p_val_adj**	**symbol**	**avg_logFC**	**p_val_adj**
Hbb-bs	-1.78511506	6.41E-05	Ctsd	1.3041957	1.56E-04	N/A	N/A	N/A	N/A	N/A	N/A
			Fcrls	0.90358352	1.60E-02						

Supplemental Experimental Procedures

Animals

9-week old male C57BL/6 mice were purchased from Charles River and maintained on a 12/12 hour light/dark cycle. Mice used in the experiments weighed between 22 and 28 g and were given food and water ad libitum. All animal experiments were approved by the Animal Care and Use Committee at the University of Virginia and conformed to the National Institutes of Health regulations for the use of animals in research.

Cationic Lipid-Shelled Microbubble Fabrication

To synthesize the cationic lipid-shelled MBs, we made a mixture of 2 mg/ml 1,2- distearoyl-sn-glycero-3-phosphocholine (DSPC; Avanti Polar Lipids, Alabastar, Alabama), 2 mg/ml polyethylene glycol 6000 monostearate (PEG 6000 MS; Stepan Kessco, Northfield, Illinois), and 0.8 mg/ml 1,2-distearoyl-3-trimethylammonium-propane (DSTAP; Avanti Polar Lipids, Alabastar, Alabama) in 0.9% NaCl (Baxter, Deerfield, Illinois). The mixture was filtered through a 0.2 um Nylon sterile filter, sparged with decafluorobutane gas (F2 Chemicals Ltd; Preston, United Kingdom), and then sonicated at the highest power (20 kHz, 30 s) with an ultrasound disintegrator (XL2020; Misonix, Farmingdale, New York) to generate the MBs. MBs were aliquoted into 13 mm glass vials, which were stoppered for storage after filling the headspace with decafluorobutane gas. The MBs were cleaned by flotation centrifugation before each experiment to remove residual micelles. An aliquot of the MB solution was centrifuged at 1000 rpm for 10 minutes, and the infranatant was removed and the bubbles resuspended in degassed saline. This process was repeated three times before the final resuspension of the bubbles at a concentration between 1.5 and 2 $x10^9$ MBs/ml. MBs were sized and counted using a Coulter counter (Multisizer 3; Beckman Coulter, Fullerton, California). When resuspended at considerably lower concentrations, MBs do not remain stable for the multiple-hour experiments.

Plasmid Preparation and Conjugation to Microbubbles

The mRuby2-N1 plasmid was a gift from Michael Davidson (Addgene plasmid #54614; http://n2t.net/addgene:54614; RRID:Addgene_54614). The plasmid features mRuby2 under control of a CMV enhancer and promoter for constitutive expression. Our studies demonstrate that peak expression is achieved by these plasmids by 24 hours after transfection and is maintained for about 1 day before beginning to decline. The plasmids was provided from Addgene (Watertown, Massachusetts) in the form of agar stabs of DH5α E. coli transformed with the plasmids. The bacteria were expanded in LB media (Sigma-Aldrich, St. Louis, Missouri) containing kanamycin (SigmaAldrich), and then the plasmids were collected and purified using an Endo-Free Maxiprep Kit (Qiagen, Germantown, Maryland). Plasmid was resuspended in Tris-EDTA buffer at a 3 concentration between 400 and 500 ng/ul and stored at -20 degrees Celsius. Plasmid concentration was determined using a NanoDrop ND-1000 spectrophotometer (NanoDrop Technologies, Wilmington, Delaware). On the morning of experiments, the appropriate purified plasmid was thawed and added to the cleaned MB solution at a ratio of 1.5 ug plasmid per 10^7 microbubbles. This ratio is consistent with prior studies of DNA binding to cationic MBs (1, 2). To achieve this, 10^9 MBs were cleaned, collected for experimentation, and combined with about 150 µg plasmid. Plasmid concentrations after Maxiprep isolation were on the order of 500 ng/µL, representing around 300 uL of plasmid solution. This mixture was allowed to incubate at room temperature for 10 min to permit the electrostatic coupling of the positively-charged bubbles and negatively-charged DNA. The final working MB concentrations were typically between 1 and 1.5 x 10^9 MBs/mL. At these concentrations, 2 x 10^5 MBs/g body weight was equivalent to roughly 3 to 5 µL of MB solution (for a 25 g mouse).

Following conjugation, a small sample of the MB-plasmid solution was collected for plasmid-binding analysis. In order to assess plasmid binding to the MBs, MBs were separated from the unbound plasmid solution by centrifugal flotation, and the infranatant was analyzed with a NanoDrop spectrophotometer to determine the unbound plasmid fraction. Roughly 25% of the incubated plasmid was bound to the MBs, for a total of 0.03 to 0.035 pg/MB, consistent with other studies in the literature investigating plasmid binding to cationic MBs. The remaining MB-plasmid solution was stored on ice. Because the solution was not post-processed by centrifugation prior to delivery to the animals, some free plasmid remained in the solution, which was accounted for in our calculations of how much additional plasmid to inject. As reported in the Supporting Information of Gorick et al. (5), no significant changes to the size distributions of the MBs after conjugation to plasmid were observed. Due to the small volume of MBs injected (a result of decreased long-term stability at lower concentrations), roughly 1 µg of plasmid was delivered in the MB-plasmid conjugate and 39 µg of free plasmid (approx. 80 µL) was injected immediately afterwards, as FUS pulsing began.

MRI-Guided FUS-Mediated Plasmid Delivery

Male C57BL/6 mice were anesthetized with an intraperitoneal injection of 120 mg/kg ketamine, 12 mg/kg xylazine, and 0.08 mg/kg atropine in sterilized 0.9% saline. A tail vein catheter was inserted to permit intravenous injections of MBs, plasmid, and the MRI contrast agent. The heads of the mice were shaved and depilated, and the animals were then placed in a supine position over a degassed water bath coupled to an MR-compatible small animal FUS system (RK-100; FUS Instruments, Toronto, Canada). The entire system was then placed in a 3T MR scanner (Magnetom Trio; Siemens Medical Solutions, Malvern, Pennsylvania). A 2-inch cylindrical transmit-receive RF coil, designed and built in-house, was placed around the mouse's head to maximize imaging SNR. Baseline T1-weighted MR images were acquired and used to select 4 FUS target locations in and around the right striatum.

Mice received an injection of the conjugated MBs (2 x 10^5 MBs/g body weight) and mRuby plasmid, followed by injection of additional free plasmid to reach a total plasmid dose of 40 ug, followed by 0.1 mL of 2% heparinized saline to clear the catheter. The total plasmid dosage of 40 ug is consistent with prior studies of cationic MB-mediated gene delivery (1 – 3). However, since we utilize a bolus injection of MBs here (as opposed to a slow infusion), we reduced the dosage of MBs to 2 x 10^5, which only allowed for a fraction of the plasmid to be delivered in MB-bound form. Thus, the injection of free plasmid immediately following the MBs was used to achieve the remainder of the 40 ug dose.

Sonication began immediately after clearance of the catheter. Sonications were performed at 0, 0.2, or 0.4 MPa PNP using a 1.1 MHz single element focused transducer operating in 10 ms bursts, 0.5 Hz pulse repetition frequency and 2 minutes total duration (i.e. 60 10 ms bursts, with 1990 ms pauses between each). These PNPs are free-field, nonderated measurements with a hydrophone in a water tank at a target distance equivalent to the treatment distance. We estimate that nonderated PNPs of 0.2 MPa and 0.4 MPa will yield PNPs of 0.16 MPa and 0.33 MPa, respectively, in brain tissue. We arrive at these estimates using values reported in the literature. It has been shown that, at frequencies ranging from 1.0 to 1.25 MHz, the middle region of the skull reduces FUS transmission no more than 20% for rats weighing between 160 and 200 g (6). In mice, attenuation has been measured to be 18% at 1.5 MHz (7). In our study, we treated mice at 1.1 MHz, for which there will be less attenuation compared to both higher frequencies (1.5 MHz) and rat skulls. Conservatively, assuming the value of 18%, 0.2 MPa and 0.4 MPa would translate to effective pressures of 0.16 MPa and 0.33 MPa, respectively.

Immediately following the FUS treatment, mice received an intravenous injection of Gd-DPTA contrast agent (0.5 µL/g body weight; Magnevist; Bayer Health Care, Indianola, Pennsylvania), and T1-weighted contrast-enhanced images were acquired to assess BBB

opening. Animals were removed from the MRI and placed on a warm pad for 30 minutes prior to reversal of the anesthetic with antisedan (1 mg/mL).

Passive Cavitation Detection

Acoustic emissions were detected with a 2.5 mm wideband unfocused hydrophone mounted in the center of the transducer. Acoustic signal was captured using a scope card (ATS460, Alazar, Pointe-Claire, Canada) and processed using an in-house built MATLAB algorithm. Acoustic emissions at the fundamental frequency, harmonics (2f, 3f, 4f), sub harmonic (0.5f), and ultra-harmonics (1.5f, 2.5f, 3.5f) were assessed by first taking the root mean square of the peak spectral amplitude (Vrms) in each frequency band after applying a 200 Hz bandwidth filter, and then summing the product of Vrms and individual sonication duration over the entire treatment period. Broadband emissions were assessed by summing the product of Vrms and individual sonication duration for all remaining emissions over the entire treatment period.

Fluorescence Activated Cell Sorting (FACS)

48 hours after FUS-treatment, mice were euthanized via CO_2 overdose. Immediately afterward, mouse brains treated with 0 MPa, 0.2 MPa, or 0.4 MPa (n = 3 per group) were harvested. The front right quadrants of each brain were excised and made into single cell suspensions using the Adult Brain Dissociation Kit (Miltenyi Biotech, Bergisch Gladbach, Germany). Suspensions were pooled by treatment group and incubated briefly with SYTOX Green Nucleic Acid Stain (1:500,000; Thermo Scientific) to identify live and dead cells. mRuby+ cells were isolated from 0.2 MPa, and 0.4 MPa cell suspensions using a BD Influx Cell Sorter (BD Biosciences, San Jose, California) with the 100 µm nozzle at 20 psi. The sort gate was established using the 0 MPa cells as a reference. Live singlet mRuby+ cells were collected for single cell RNA-sequencing. FACS data were analyzed using FCS Express 6 software.

Supplemental References

1. J. P. Christiansen, B. A. French, A. L. Klibanov, S. Kaul, J. R. Lindner, Targeted tissue transfection with ultrasound destruction of plasmid-bearing cationic microbubbles. *Ultrasound Med. Biol.* **29**, 1759–1767 (2003).
2. A. R. Carson, *et al.*, Gene therapy of carcinoma using ultrasound-targeted microbubble destruction. *Ultrasound Med. Biol.* **37**, 393–402 (2011).
3. C. M. Panje, *et al.*, Ultrasound-Mediated Gene Delivery with Cationic Versus Neutral Microbubbles: Effect of DNA and Microbubble Dose on *In Vivo* Transfection Efficiency. *Theranostics* **2**, 1078–1091 (2012).
4. Franzén, Oscar, Li-Ming Gan, Johan LM Björkegren, PanglaoDB: a web server for exploration of mouse and human single-cell RNA sequencing data. *Database* 2019 (2019).
5. Gorick CM, Mathew AS, Garrison WJ, Thim EA, Fisher DG, Copeland CA *et al.* Sonoselective transfection of cerebral vasculature without blood–brain barrier disruption. *Proc Natl Acad Sci*. **117**, 5644–5654 (2020).
6. Gerstenmayer, Matthieu, *et al.*, Acoustic transmission factor through the rat skull as a function of body mass, frequency and position. *Ultrasound Med. Biol.* **44**, 2336-2344 (2018).
7. Choi, James J., *et al.*, Noninvasive, transcranial and localized opening of the blood-brain barrier using focused ultrasound in mice. *Ultrasound Med. Biol.* **33**, 95-104 (2007).

R Session Info

```
R version 4.0.0 (2020-04-24)
Platform: x86_64-w64-mingw32/x64 (64-bit)
Running under: Windows 10 x64 (build 19041)

Matrix products: default

Random number generation:
 RNG:     Mersenne-Twister
 Normal:  Inversion
 Sample:  Rounding

locale:
[1] LC_COLLATE=English_United States.1252  LC_CTYPE=English_United States.1252    LC_MONETARY=English_United States.1252
[4] LC_NUMERIC=C                           LC_TIME=English_United States.1252

attached base packages:
[1] parallel  stats4    stats     graphics  grDevices utils     datasets  methods   base

other attached packages:
 [1] pander_0.6.3                metap_1.4                   assertive.types_0.0-3       magrittr_1.5
 [5] egg_0.4.5                   gridExtra_2.3               ggnomics_0.1.1              ggforce_0.3.1
 [9] svglite_1.2.3               BBmisc_1.11                 scales_1.1.1                Rmosek_1.3.5
[13] DT_0.13                     ashr_2.2-47                 apeglm_1.10.0               data.table_1.12.8
[17] limma_3.44.1                tximportData_1.16.0         plyr_1.8.6                  tximport_1.16.0
[21] ggrepel_0.8.2               MAST_1.14.0                 SingleCellExperiment_1.10.1 DESeq2_1.28.1
[25] SummarizedExperiment_1.18.1 DelayedArray_0.14.0         matrixStats_0.56.0          Biobase_2.48.0
[29] GenomicRanges_1.40.0        GenomeInfoDb_1.24.0         IRanges_2.22.2              S4Vectors_0.26.1
[33] BiocGenerics_0.34.0         Tmisc_0.1.22                fgsea_1.14.0                forcats_0.5.0
[37] stringr_1.4.0               purrr_0.3.4                 readr_1.3.1                 tidyr_1.1.0
[41] tibble_3.0.1                tidyverse_1.3.0             annotables_0.1.91           ggplot2_3.3.1
[45] Seurat_3.1.5.9008           dplyr_1.0.0                 biomaRt_2.44.0

loaded via a namespace (and not attached):
  [1] reticulate_1.16           tidyselect_1.1.0          RSQLite_2.2.0             AnnotationDbi_1.50.0
  [5] htmlwidgets_1.5.1         grid_4.0.0                BiocParallel_1.22.0       Rtsne_0.15
  [9] munsell_0.5.0             mutoss_0.1-12             codetools_0.2-16          ica_1.0-2
 [13] future_1.17.0             withr_2.2.0               colorspace_1.4-1          rstudioapi_0.11
 [17] ROCR_1.0-11               assertive.base_0.0-7      gbRd_0.4-11               listenv_0.8.0
 [21] Rdpack_2.0                bbmle_1.0.23.1            GenomeInfoDbData_1.2.3    mnormt_2.0.2
 [25] mixsqp_0.3-43             polyclip_1.10-0           farver_2.0.3              bit64_0.9-7
 [29] TH.data_1.0-10            coda_0.19-3               vctrs_0.3.0               generics_0.0.2
 [33] xfun_0.14                 BiocFileCache_1.12.0      R6_2.4.1                  rsvd_1.0.3
 [37] invgamma_1.1              locfit_1.5-9.4            bitops_1.0-6              assertthat_0.2.1
 [41] multcomp_1.4-14           gtable_0.3.0              globals_0.12.5            sandwich_3.0-0
 [45] rlang_0.4.6               genefilter_1.70.0         systemfonts_0.2.2         splines_4.0.0
 [49] lazyeval_0.2.2            broom_0.5.6               checkmate_2.0.0           reshape2_1.4.4
 [53] abind_1.4-5               modelr_0.1.8              backports_1.1.7           tools_4.0.0
 [57] ellipsis_0.3.1            RColorBrewer_1.1-2        ggridges_0.5.2            TFisher_0.2.0
 [61] Rcpp_1.0.4.6              progress_1.2.2            zlibbioc_1.34.0           RCurl_1.98-1.2
 [65] prettyunits_1.1.1         openssl_1.4.1             pbapply_1.4-2             cowplot_1.0.0
 [69] zoo_1.8-8                 haven_2.3.0               cluster_2.1.0             fs_1.4.1
 [73] tinytex_0.23              lmtest_0.9-37             reprex_0.3.0              RANN_2.6.1
 [77] tmvnsim_1.0-2             truncnorm_1.0-8           mvtnorm_1.1-0             SQUAREM_2020.2
 [81] fitdistrplus_1.1-1        hms_0.5.3                 patchwork_1.0.0           xtable_1.8-4
 [85] XML_3.99-0.3              emdbook_1.3.12            readxl_1.3.1              compiler_4.0.0
 [89] bdsmatrix_1.3-4           KernSmooth_2.23-17        crayon_1.3.4              htmltools_0.4.0
 [93] geneplotter_1.66.0        lubridate_1.7.8           DBI_1.1.0                 tweenr_1.0.1
 [97] dbplyr_1.4.4              MASS_7.3-51.6             rappdirs_0.3.1            Matrix_1.2-18
[101] cli_2.0.2                 rbibutils_1.3             igraph_1.2.5              pkgconfig_2.0.3
[105] sn_1.6-2                  numDeriv_2016.8-1.1       plotly_4.9.2.1            xml2_1.3.2
[109] annotate_1.66.0           multtest_2.44.0           XVector_0.28.0            rvest_0.3.5
[113] digest_0.6.25             sctransform_0.2.1         RcppAnnoy_0.0.16          cellranger_1.1.0
[117] leiden_0.3.3              fastmatch_1.1-0           uwot_0.1.8                gdtools_0.2.2
[121] curl_4.3                  lifecycle_0.2.0           nlme_3.1-148              jsonlite_1.6.1
[125] viridisLite_0.3.0         askpass_1.1               fansi_0.4.1               pillar_1.4.4
[129] lattice_0.20-41           plotrix_3.7-8             httr_1.4.1                survival_3.1-12
[133] glue_1.4.1                png_0.1-7                 bit_1.1-15.2              assertive.properties_0.0-4
[137] stringi_1.4.6             blob_1.2.1                memoise_1.1.0             mathjaxr_1.0-1
[141] irlba_2.3.3               future.apply_1.5.0        ape_5.3
```
